# Supplementary material for: A new analysis method for evaluating bacterial growth with microplate readers
Source: PLoS One. 2021 Jan 12;16(1):e0245205. doi: 10.1371/journal.pone.0245205 (PMC7802944; doi:10.1371/journal.pone.0245205)
Supplement: S1 File — (PDF) [file pone.0245205.s001.pdf]

A new analysis method for evaluating bacterial growth with microplate readers

Venkata Rao Krishnamurthi [1], Isabelle I. Niyonshuti [2], Jingyi Chen [2,3], Yong Wang [1,3,4,*]

[1] Department of Physics, [2] Department of Chemistry and Biochemistry, [3] Materials Science and Engineering Program, [4] Cell and Molecular Biology Program, University of Arkansas, Fayetteville, AR 72701.

* To whom correspondence should be addressed: yongwang@uark.edu (Y.W.)

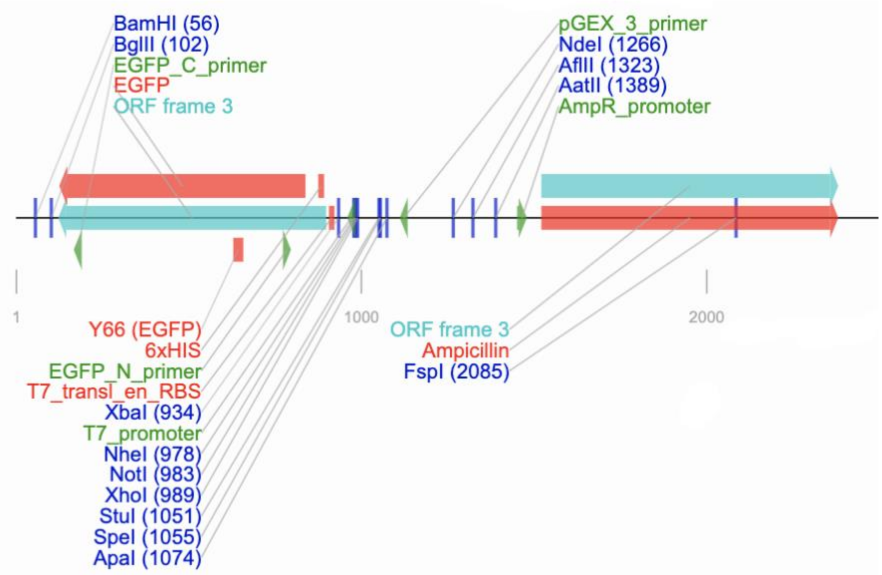

Figure S1. Sequence map of the segment of the plasmid involving the enhanced GFP and ampicillin. The sequence of the plasmid segment was shared by Dr. David McMillen's research group (see the Acknowledgment). The sequence map was generated by the Sequence Analyzer tool from Addgene (http://www.addgene.org/analyze-sequence/).

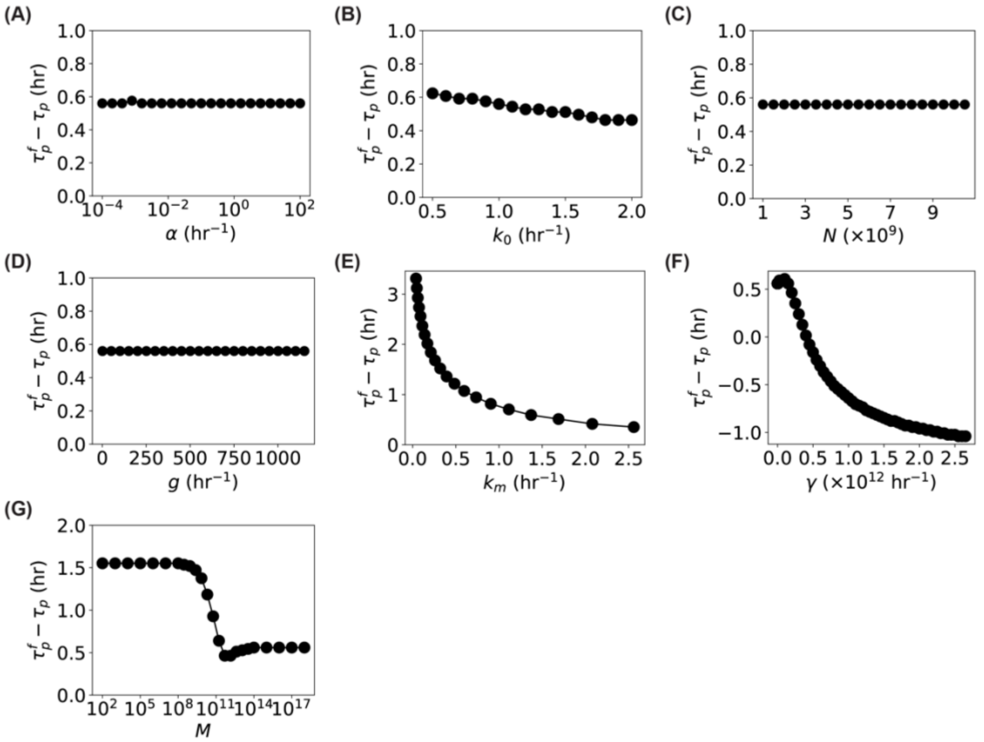

Figure S2. Dependence of the difference in τ_p^f and τ_p (i.e., the horizontal shift observed in Fig. 2F) on the various parameters: (A) activation rate α, (B) maximum growth rate k_0, (C) maximum possible number of bacteria N, (D) fluorescent protein expression/generation rate g, (E) maturation rate k_m, (F) degradation rate γ, and (G) degradation capacity M.

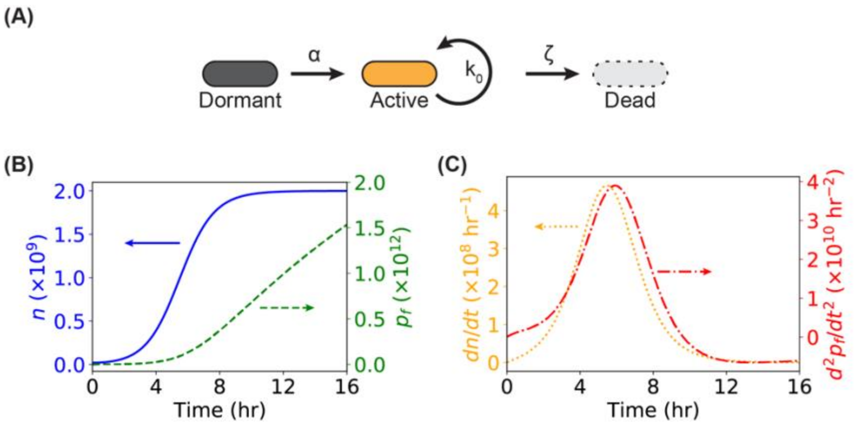

Figure S3. (A) A model considering cell death with a death rate of ζ. The Eq. (2) is changed to $\frac{dn_A}{dt} = +\alpha n_D + k_0\left(1 - \frac{n_A + n_C}{N}\right)n_A - \zeta n_A$, where n_C is the number of dead (ceased) bacteria following $\frac{dn_C}{dt} = +\zeta n_A$. (B) Predictions from the updated model with the same parameters as in Fig. 2 and $\zeta = 0.05$ hr^{-1}.
