# Supplementary material for: A new analysis method for evaluating bacterial growth with microplate readers
Source: PLoS One. 2021 Jan 12;16(1):e0245205. doi: 10.1371/journal.pone.0245205 (PMC7802944; doi:10.1371/journal.pone.0245205)
Supplement: Attachment — Submitted filename: rebuttal_lett.pdf [file pone.0245205.s002.pdf]

Dear Editor,

Thank you for handling our manuscript PONE-D-20-34200. We appreciate the reviewers’ time and effort for reviewing our manuscript and providing constructive, helpful comments. In this rebuttal/response letter, we address the reviewers’ comments in details point-by-point. In addition to the revised clean (unmarked) manuscript, a marked-up version highlighting the revisions is also uploaded at the resubmission. We hope that the revised article is now suitable for publication in *PLOS ONE*. Thank you.

Sincerely,

Yong Wang
(on behalf of the authors)

Reviewer #1

The manuscript by Krishnamurthi et al is an interesting work that relates to the general working in a microbiology laboratory. The method developed here allows one to estimate the lag time in the growth of a bacterium, which can be used for comparison between conditions such as, for example, normal and toxic. While the work and the analyses are generally good, there are several issues with the paper that I describe below.

Author Response: We thank the reviewer for the positive comments.

1. Lines 58 - 60. “First, multiple scattering of microbes is more severe and problematic in microplate reader-based OD measurements than in cuvette-based ones (2)” - The reference given does not provide any information to support this statement. Multiple scattering is a function of cell density in the sample. The error introduced by multiple scattering, however, depends on factors such as aperture radius and the distance between detector and sample, etc. of the instrument used. The cited paper, however, does not suggest anything about how these factors vary between plate readers and conventional cuvette-based experiments. The authors only claim that since plate readers are predominantly used for measurements at higher culture densities, there are errors in estimating cell numbers, which, I believe, is true even for cuvette-based experiments.

Author Response: The reviewer is correct, and the statement in the original manuscript was unclear and misleading. We have revised the statement following the reviewer’s suggestion. We thank the reviewer for the constructive comment. In the resubmission, it reads the following (L58-60).

> “... First, multiple scattering of microbes is usually severe and problematic in microplate reader-based OD measurements, as microplate readers have been predominantly used at higher culture densities (2). ...”

2. Line 123. It is not clear why Pn (number of non-fluorescent proteins) is used in the numerator of the second part of the RHS of the equation “P/ = kP − P/(P+P+).” It should have been P instead of P since this part of the equation accounts for the degradation of fluorescent GFP proteins in the system.

Author Response: We thank the reviewer for catching the typo: the numerator of the last term in Eq. (4) should be γP_f. We also confirm that, by double-checking the simulation code, the correct equation was used in all the simulations. We have corrected this typo in the revised manuscript.

3. Lines 223-224. “We note that there is a small horizontal shift between the two peaks (Fig. 2F)”. – Here, this shift between the peaks of OD and fluorescence derivate curves is not further explored, particularly if the shift is a function of any of the growth or fluorescent protein expression parameters. If it is so, comparing this peak location across samples where these parameters vary could produce errors.

Author Response: First, we would like to point out that the horizontal shift was further explored in our original manuscript (for example in Fig. 3E-3F, Fig. 4F, Fig. 5B, Fig. 5D, Fig. 6B, and

Fig. 6D where we showed τ_p^f and τ_p [if τ_p varies]). Yet, we acknowledge that we failed to explicitly highlight our investigation on this horizontal shift throughout the manuscript. We have added several sentences to explicitly emphasize further investigations on the horizontal shift (L229-230, L250-254, L290-291, L305-306, L313-316, L340-342).

Second, we agree with the reviewer that the horizontal shift is mainly due to the parameters related to the fluorescent proteins. As shown in Fig. 3E and 4F, the differences between τ_p^f and τ_p remained almost constant when the activation rate α or the maximum growth rate k_0 changed. In contrast, varying the maturation rate k_m or the degradation rate γ resulted in significant changes in τ_p^f (Fig. 5D and 6B) while τ_p remained constant, thus leading to significant changes in the horizontal shift ($\tau_p^f - \tau_p$). A systematic comparison showed that this horizontal shift depends mostly on k_m, significantly on γ and M, slightly on k_0, but not on α, N, and g (see Fig. R1 below). We have included Fig. R1 as a supplementary figure (Fig. S2) in the Supporting Information and added one paragraph in the Discussion section of the resubmitted manuscript (L434-440).

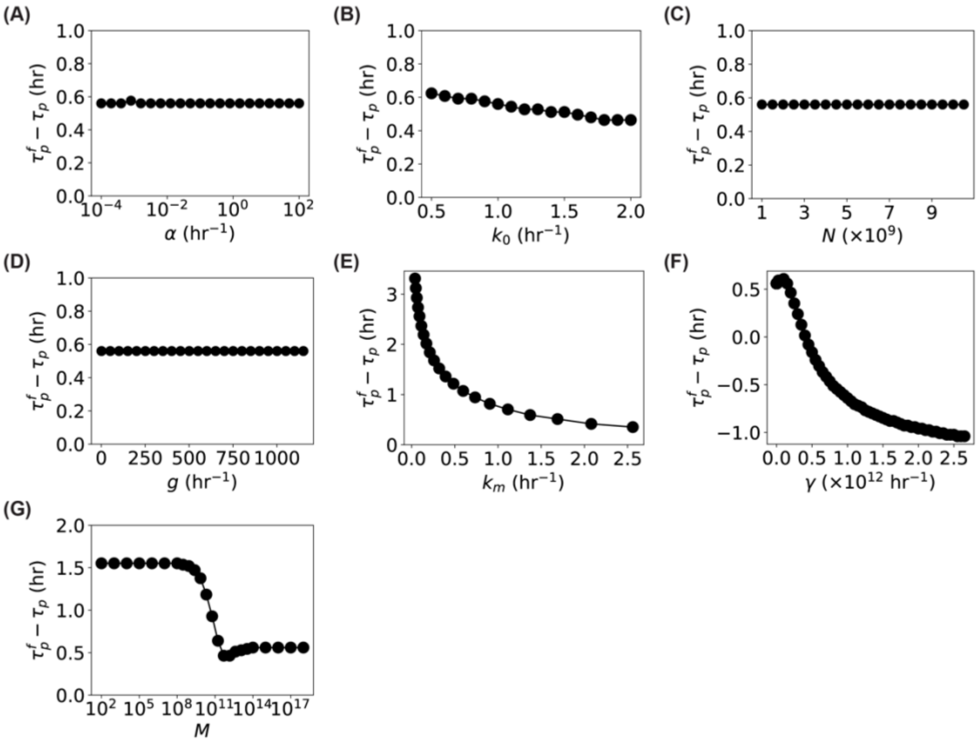

Figure R1. Dependence of the difference in τ_p^f and τ_p (i.e., the horizontal shift observed in Fig. 2F) on the various parameters: (A) activation rate α, (B) maximum growth rate k_0, (C) maximum possible number of bacteria N, (D) fluorescent protein expression/generation rate g, (E) maturation rate k_m, (F) degradation rate γ, and (G) degradation capacity M.

It is also worthwhile to emphasize that the dependencies of the changes in the peak location (horizontal shift) and peak height (vertical shift) on the various growth parameters (α, k_0, N, g,

k_m, γ, M) are many-to-many mapping. In other words, a change in the peak location or height can possibly depend on several parameters, and one parameter can possibly affect changes in both the peak location and height. On one hand, we agree with the reviewer that this many-to-many relationship brings complications if one wants to compare the growth rate (related to k_0) and lag time (related to α) from the fluorescence-based peaks among samples in which the fluorescence-related parameters vary. On the other hand, we would like to note that the many-to-many mapping could be an advantage instead of a problem in other scenarios and in general. First, it is not a problem because one can use the location (τ_p) and height (η_p) of the peak derived from the OD-based growth curves for comparisons among samples if the growth rate and lag time are the parameters of interest. As stated in the manuscript, the parameters related to the fluorescent proteins (g, k_m, γ, M) do not affect the location and height of the peaks derived from the OD-based growth curves. It is important to clarify that it is highly recommended to use the time derivatives from both the OD and fluorescence growth curves (as we did in Fig. 7). Second, as discussed in the paper, the major purpose of developing the current method is not to replace the traditional OD measurements and mathematical fittings of growth curves. Instead, it is a complementary means to assess the growth behaviors of microbes. We do ***not*** claim that one should only use the fluorescence growth curves, nor that the time derivatives from the fluorescence curves are sufficient for complete descriptions of the bacterial growth. What we meant is that, if it is not practical to obtain "good" OD curves, the fluorescence-based growth curves and their derivatives provide a new way to assess the growth behaviors and properties of the bacteria and their changes. Third, as the expression and maturation of fluorescent proteins are also important aspects of the growth behaviors of microbes, the peak properties of the fluorescence-based growth curves provide ***additional*** reporters for quantitative evaluating the growth of microbes. Fourth, the current method can be very useful in applications and situations where toxins or reagents cause different responses in the OD-based and fluorescent-based growth curves. One of such examples is that polydopamine causes significant changes in the fluorescent-based growth curves but has much less effects on the OD-based growth curves (Cong et al., 2014; Niyonshuti et al., 2020). These discussions were added in the revised manuscript (L428-432 and L442-452)

4. Although the authors claim that the first derivative of growth curve can be used in calculating growth parameters from samples that do not fit into traditional sigmoid curves due to multiple scattering, none of the modelling results justify why this can be done. At high multiple scattering regimes, OD is expected to have a parabolic dependency on bacterial number, N (see your reference 2). It is not clear why taking first derivative should eliminate the non-linear cell concentration dependent contribution of multiple scattering to OD. None of the stimulations performed has included this contribution from multiple scattering. Calculating growth parameters from derivative curves of non-sigmoid growth profiles when the relationship between OD curve and its derivate itself was derived using a simple sigmoid growth model seems erroneous.

Author Response: The reason why taking the first-order time derivative of the OD-based growth curve helps to solve the problem of multiple scattering (or nonlinear OD-concentration relation) can be understood by the following argument.

(1) Multiple scattering typically leads to flattening/saturation of the growth curve, showing lower slopes, which can be seen from Fig. 1C of Ref. 2 (Stevenson et al., 2016) and Fig. 7A of our manuscript.
(2) Lower slopes in the OD curve will result in lower values when taking the first derivative of the curve, which will appear as shoulders or long tail of the peak (e.g., Fig. 7C).
(3) The long tail or shoulder of the peak does not play a role when identifying the peak location; therefore, the effect of the multiple scattering on the OD curve is lessened or (partly) eliminated by taking the first derivative of the OD growth curve.

Note that we chose to use and examine the cell number $n(t)$, instead of the OD (or transmittance), in all our simulated results. This choice was made because the relation between the cell number (or concentration) and the OD (or transmittance) is complicated when considering multiple scattering and instrument parameters (e.g., aperture shape, aperture radius, and the distance between detector and sample). In certain regimes, the parabolic dependence of the OD on cell number, mentioned by the reviewer, is valid, while it is not in general. As the Koch stated in his original paper where he proposed the use of parabolas, "the choice of a second-degree polynomial with no constant term to fit the data is arbitrary" (Koch, 1970), and the parabolic relation is only valid for "ideal instrumentation and nonabsorbing bacteria" (Koch, 1970). Because the main purpose of the current manuscript is to introduce the usage of the time-derivatives of the OD-based and fluorescence-based growth curves, we feel that it is out of the scope of the current work to perform exact and detailed simulations for converting cell numbers to OD values while taking into account multiple scattering and instrument parameters.

To improve the clarity of the manuscript about this concern brought up by the reviewer, we added several paragraphs in the Discussion section (L464-482). We thank the reviewer for helping us to improve our manuscript.

5. Since it is shown in the simulations that the peak location and peak height of the second derivative of fluorescence curves is dependent on parameters such as GFP expression rate, maturation rate, degradation rate, etc., it should be noted in the limitations part that the use of fluorescent readings can be used to estimate cell density across different samples only if these parameters for fluorescence protein concentration remain constant throughout the samples compared. One of the most important limitations of the proposed strategy could be that the value obtained from the second derivative graph of fluorescence, namely peak location is dependent on multiple independent growth factors such as activation rate and maximum growth rate. Therefore the peak location obtained from graphs of different samples can only be used to quantify the change in one of the growth parameters if one makes sure that the other parameter remains constant. This is not the case most of the time.

Author Response: On one hand, we agree with the reviewer that the dependencies of peak location and height on the parameters related the fluorescent proteins bring complications and limitations as described by the reviewer. On the other hand, we would like to point that the dependencies could be advantages in certain situations, as we pointed out in our response to Major Comment 3 (please see above). In the revised manuscript, we have added discussions

related to this comment at various places throughout the manuscript, including the Discussion section (L428-452).

Other minor comments:
1. It is not clear how GFP expresses - from a constitutive promoter or induced? I was unable to find details regarding the plasmid even from the cited reference.

Author Response: The enhanced GFP (EGFP) proteins were expressed continuously with a constitutive T7 promoter. Induction was not needed nor used. The sequence map of the segment of the plasmid for the enhanced GFP (EGFP) and ampicillin is shown below (Fig. R2, the same as Fig. S1). We have added more descriptions of the plasmid in the Materials and Methods of the revised manuscript (L166-169, and L176-178).

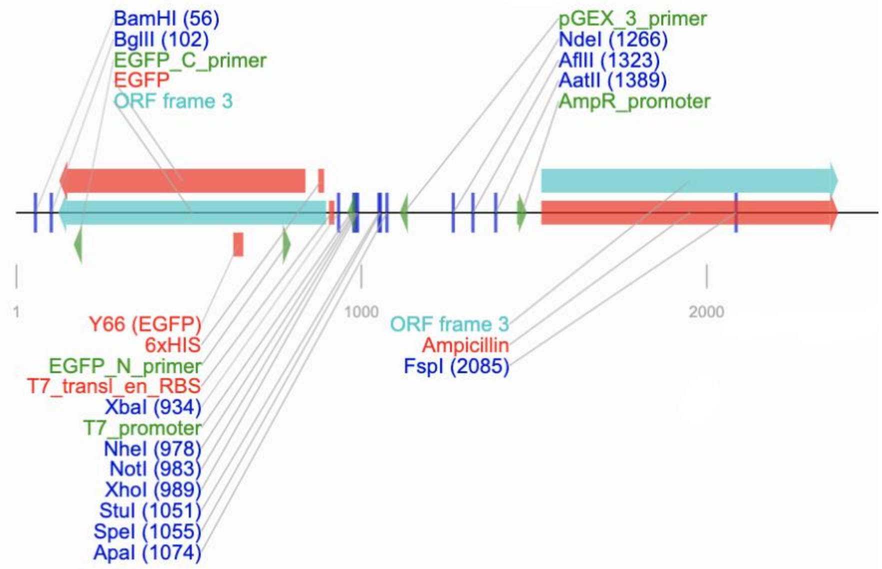

Figure R2. Sequence map of the segment of the plasmid involving the enhanced GFP and ampicillin. The sequence of the plasmid segment was shared by Dr. David McMillen's research group (see the Acknowledgment). The sequence map was generated by the Sequence Analyzer tool from Addgene (http://www.addgene.org/analyze-sequence/).

2. Is it possible that the GFP expression can directly be affected by the added Ag? That will also lead to errors in lag time calculation. Further, if added Ag affects plasmid replication, this will be an issue. In such scenario, it would be preferred to have an integrated plasmid expressing GFP.

Author Response: In the current work, we are not able to exclude the possibility that the expression of GFP is directly affected by the addition of silver ions or nanoparticle. However, this possibility is low because the Ag^+-caused changes in peak locations estimated from the OD-based growth curves and fluorescence-based growth curves were consistent with each other (i.e., $\Delta\tau_p = \Delta\tau_p^f$ as shown in Fig. 7).

In addition, we would like to clarify and emphasize that it is highly recommended to use the time derivatives from both the OD and fluorescence growth curves (as we did in Fig. 7) with the current method. both OD and fluorescence curves should be used, when taking time derivatives

and assessing growth properties of the bacteria, in the current method whenever possible (i.e., as what we did in Fig. 7). We do *not* claim that one should only use the fluorescence growth curves, nor that the time derivatives from the fluorescence curves are sufficient for complete descriptions of the bacterial growth. What we meant is that, if it is not practical to obtain "good" OD curves, the fluorescence-based growth curves and their derivatives provide a new way to assess the growth behaviors and properties of the bacteria and their changes. Furthermore, as the expression and maturation of fluorescent proteins are also important aspects of the growth behaviors of microbes, the peak properties of the fluorescence-based growth curves provide *additional* reporters for quantitative evaluating the growth of microbes. Therefore, we would like to argue that it is an advantage and/or feature of the current method to be capable of detecting changes in the bacterial growth due to changes in protein expression.

Such discussions were added to the resubmitted manuscript (L428-452).

3. One of the outcomes of the growth curve is the mean generation time. This is not possible with the method developed here.

Author Response: We do not agree with the reviewer: it is possible to estimate the changes in the mean generation time from the current method because (1) the mean generation time (T_d) is related to the growth rate k_0, $T_d = \frac{\ln 2}{k_0} \propto \frac{1}{k_0}$ (Juska et al., 2006); and (2) η_p is linearly dependent on and *only* on k_0 (Fig. 4). Therefore, from the changes in the peak height of the time derivative of OD-based curves, it is possible to estimate the change in the mean generation time. On the other hand, we acknowledge that, if only the fluorescence-based growth curves are available, it is practically difficult to quantify the mean generation time because, in addition to k_0, both τ_p^f and η_p^f are also coupled to the parameters related to fluorescent proteins. We have added a paragraph of discussion in the revised text (L454-462).

Reviewer #2

The manuscript by Krishnamurthi et al showed a new time derivative method for evaluating the bacterial growth. The precise evaluation on the growth rate is highly required for better understanding of growing cells and populations. Authors have beautifully targeted and addressed a very the basic challenge pertaining to the evaluation of the bacterial growth. Some of the questions and queries that came across are as follows:

Author Response: We really appreciate the highly positive comments from the reviewer.

1. Authors should re-check the equation 4 on page 5 of the manuscript. In my opinion second term on right hand site should be degradation of number of fluorescent proteins. Or if there is any specific reason for inclusion of the said term, it should be specified.

Author Response: We thank the reviewer for catching the typo: the numerator of the last term in Eq. (4) should be γP_f. We also confirm that, by double-checking the simulation code, the correct equation was used in the simulations. We have corrected this typo in the revised manuscript.

2. Generally Optical density-based methods requires high bacteria concentrations (about 10^7 cells/ml). Since current method is based on the time derivatives of optical density, the robustness of the method needs to be mentioned in terms of bacterial concentrations especially at lower concentrations.

Author Response: We agree with the reviewer that OD based methods typically has a lower limit of 0.01-0.02 (corresponding to ~10^7 cells/ml). This is one of the reasons why the initial cell number is 2×10^7 in our simulations. The robustness of the current method relies on the sensitivity and robustness of the instrument for absorbance/OD and fluorescence measurements. Therefore, the current method of taking the time derivative of the OD curves also requires high bacteria concentrations. We have added a short comment in the revised text (L424-426).

3. Further, considering the fact that more robust, time-lapse bright field imaging system are available that enable rapid higher throughput, non-invasive, real-time monitoring of microbial growth and morphological features, how current method resolves the challenge of differentiating the live and dead cells based on OD or FL.

Author Response: We thank the reviewer for bringing up this issue. The current method is based on OD and FL with microplate readers and thus reports the growth properties of bacteria at the ensemble level (i.e., the average over many bacteria). Therefore, the current method cannot differentiate live and dead cells, which is a limitation compared to time-lapse microscopy. We previously used time-lapse microscopy to investigate the growth of bacteria at the single-cell level, which was able to differentiate live and dead cells, measure the growth of individual cells, and monitor the morphological changes of bacteria (Krishnamurthi et al., 2019). However, based on our experience, time-lapse microscopy is not as convenient as the microplate-reader based method in certain situations (especially high throughput is desired); it has its own limitations, such as low throughput, defocusing over long time period, stage drift, limited size of field of view (FOV), variance among different FOVs, etc., although some of the limitations can be

overcome by exploiting other techniques (such as microfluidics, automated scanning of FOVs, and drift correction). Therefore, the different methods have different advantages and different applications. It is also worthwhile to note that there exist modern microplate readers that combines time-lapse imaging with growth curve measurements, which combines the advantages from both types of measurements.

In the resubmitted manuscript, we have added a paragraph of discussion (L504-L516).

4. How about the versatility of this model? Is this method applicable to other methods such as colony forming unit (CFU) and ATP abundance-based methods used to monitor bacterial growth?

Author Response: The reviewer brings up a very interesting aspect. We have not done any investigations on the applications of the current time-derivative method on CFU assays or ATP abundance-based methods. We expect that the current method is *not* suitable for CFU assays because our method requires reasonably high temporal resolution (i.e., the time intervals of data points should be shorter than the dynamics of interest), while CFU assays typically do not have good temporal resolutions. On the other hand, the current method is likely applicable to ATP abundance-based assays as the temporal resolution could be high enough. It would be interesting to experimentally demonstrate the time-derivative method on ATP-based assays in the future. Frankly, we are also inspired by the reviewer that a potential future study is to extend the current time-derivative method to microbiological/biochemical assays for other purposes (such as metabolism and enzymatic activities).

We added a paragraph of discussions for this comment in the revised manuscript (L534-542).

5. Recently, Optical Density and fluorescence based more robust methods of measuring bacterial growth by simple calibrations using silica microsphere have been proposed (Beal, J., Farny, N.G., Haddock-Angelli, T. et al. Robust estimation of bacterial cell count from optical density. Commun Biol 3, 512 (2020). https://doi.org/10.1038/s42003-020-01127-5). What are the advantages of your method over these minute calibrations?

Author Response: We thank the reviewer for mentioning the paper (Beal et al., 2020), in which different calibration methods were compared. The authors concluded that serial dilution of silica microspheres worked the best for easy, accurate, and robust calibration of an instrument for converting OD to cell numbers. We believe that this beautiful interlaboratory study (Beal et al., 2020) will be very useful in the field of microbiology.

We note that our work offered an alternative method *without* the use of calibrations, introduced the use of the time-derivatives of the growth curves, and demonstrated how this method could be applied to quickly identify changes in the growth properties of bacteria. In addition, the current method does not exclude the use of growth curve measurements that are carefully calibrated following recent works (Beal et al., 2020; Stevenson et al., 2016). We believe that these accurate and robust calibration methods provide better data to start with for analysis using the current method.

A brief discussion was added into the revised text (L484-491).

Minor Comments:
1. Conclusion section needs to be shortened

Author Response: We have followed the reviewer's suggestion and shortened the Conclusion section by moving most of the discussions to a separate Discussion section.

Reviewer #3

The authors in this study aims to develop a method for evaluating the growth of bacteria measured with multimode microplate readers based on the time derivatives of the OD and/or FL of the bacteria. Moreover, they conclude that their methods provides a better way for interpreting OD growth curves when the growth curve does not follow the well-established sigmoid shape due to multiple scattering. The work is good and the paper is will written. However, there are some minor points that need to be warranted before the publication of this manuscript.

Author Response: We thank the reviewer for the positive comments.

• The authors have tested their hypothesis on bacteria expressing Green Fluorescent Protein. This implies that the method is applicable to only those bacterial population expressing GFP. If we are working with some pathogenic bacteria which don't express GFP, then will this method work?

Author Response: We feel that there might exist confusion about the current method and would like to clarify that the current method focuses on the use and interpretation of the time derivatives of the growth curves. In the current method, the time derivatives of both the OD and fluorescence curves could *and* should be used, as we did in Fig. 7. Whenever possible, it is highly recommended to use both OD and fluorescence growth curves. We do *not* claim that one should only use the fluorescence growth curves, nor that the time derivatives from the fluorescence curves are sufficient for a complete description of the bacterial growth. What we meant is that, if it is not practical to obtain "good" OD curves, the fluorescence-based growth curves and their derivatives provide a useful way to assess the growth behaviors and properties of the bacteria and their changes.

Therefore, we expect that the current method can be applied to bacteria that do not express GFP by simply taking time derivatives on the OD growth curves. It can be also applied to bacteria expressing fluorescent proteins other than GFP.

Clarifications were added into the revised manuscript in the Discussion section (L428-452, and L534-542).

• Since this method works with bacteria having GFP, the authors assume that as bacterial cell divides, the GFP molecule will be passed on to the daughter cells. However, this is not true and many times plasmid is lost from daughter cells if they are not grown in selective media. Have the authors taken this into the account?

Author Response: We agree with the reviewer that, in general, plasmid loss could be a potential problem especially when selection pressure is absent. However, media with antibiotics are used in many (if not most) laboratory-scenarios, so that the plasmids are not lost over generations in bacteria. In addition, we note that the plasmid used in the current study is a high-copy number plasmid, and that antibiotic was used; therefore, we expect that the plasmids were maintained well in the bacteria. We have added a brief comment in the revised manuscript (L508-516).

• During the growth phase of the bacteria, death of bacterial cells also occur. Does the authors have included death parameter as well in the equations and experimentation process?

Author Response: We did not include cell death in the simulations in the original manuscript. One reason is that cell death is rare in growing cultures of bacteria (e.g., *E. coli*) maintained under constant conditions (i.e., typically laboratory-scenarios) (Koch, 1959). Also, as noted in the discussions, we adopted a very simple model for the growth of bacteria and expression of fluorescent proteins in the current work, but there are better models available in the literature (Haque et al., 2017; Klanjscek et al., 2012, 2013), including one of our own published papers (Haque et al., 2017). Klanjscek et al. (from Dr. Holden's group) have done beautiful works and developed detailed models that involve cell death caused by toxins and toxicants (Klanjscek et al., 2012, 2013). We note that it is not our focus in the current work to develop models to include all the details related to bacterial growth. The main purpose of the model in this manuscript is to help to explain why the current method works and to interpret the results at the basic level.

On the other hand, to address the reviewer's concern, we performed more simulations, in which bacteria will die with a death rate of ζ (Fig. R3A). Then Eq. (2) is changed to

$$\frac{dn_A}{dt} = +\alpha n_D + k_0\left(1 - \frac{n_A+n_C}{N}\right)n_A - \zeta n_A \quad \text{(R-E1)}$$

where n_C is the number of dead (ceased) bacteria, which follows

$$\frac{dn_C}{dt} = +\zeta n_A \quad \text{(R-E2)}$$

To quantitative estimate the effect of cell death, we took $\zeta = 5\%$ hr^{-1}. Note that ζ is expected to be much smaller than 5% hr^{-1} under normal growth conditions; 5% hr^{-1} corresponded to the death rate of bacteria when the bacteria were chilled and centrifuged (Koch, 1959). We observed very similar growth curves and time derivatives (Fig. R3B). Therefore, the main conclusions of the manuscript were not affected.

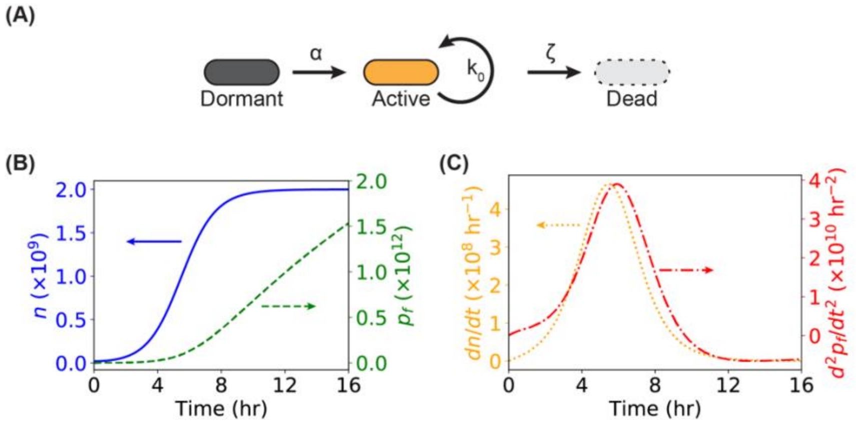

Figure R3. (A) Updated model when considering cell death with a death rate of ζ. (B) Predictions from the updated model with the same parameters as in Fig. 2 and $\zeta = 0.05$ hr^{-1}.

We have included the new simulations, results, and figure (Fig. S3) in the Supporting Information. We also added more comments in the Discussion (L508-516).

• There are some reports linking the quality of GFP expression and bacterial growth. Have the authors taken account of this fact in their study?

Author Response: We did not explicitly take into account the relation between the quality of GFP expression and bacterial growth. If the relations are explicitly available, we can include the parameters related to the fluorescent proteins as functions of bacterial growth parameters in the models, i.e., $g(\alpha, k_0)$, $k_m(\alpha, k_0)$, etc. However, we don't think it is within the scope of the current work to further explore in this direction. The major purpose of the current work is to introduce the use of the time-derivatives of the growth curves and demonstrated how this method could be applied to identify changes in the growth properties of bacteria. The current method is limited in identifying which parameter is responsible for the change.

We have added a brief comment in the revised manuscript (L508-516).

References

Beal, J., Farny, N.G., Haddock-Angelli, T., Selvarajah, V., Baldwin, G.S., Buckley-Taylor, R., Gershater, M., Kiga, D., Marken, J., Sanchania, V., *et al.* (2020). Robust estimation of bacterial cell count from optical density. Communications biology *3*, 512-512.

Cong, Y., Xia, T., Zou, M., Li, Z.N., Peng, B., Guo, D.Z., and Deng, Z.W. (2014). Mussel-inspired polydopamine coating as a versatile platform for synthesizing polystyrene/Ag nanocomposite particles with enhanced antibacterial activities. Journal of Materials Chemistry B *2*, 3450-3461.

Haque, M.A., Imamura, R., Brown, G.A., Krishnamurthi, V.R., Niyonshuti, I.I., Marcelle, T., Mathurin, L.E., Chen, J., and Wang, Y. (2017). An experiment-based model quantifying antimicrobial activity of silver nanoparticles on Escherichia coli. RSC Advances *7*, 56173-56182.

Juska, A., Gedminiene, G., and Ivanenc, R. (2006). Growth of microbial populations. Biochemistry and Molecular Biology Education *34*, 417-422.

Klanjscek, T., Nisbet, R.M., Priester, J.H., and Holden, P.A. (2012). Modeling physiological processes that relate toxicant exposure and bacterial population dynamics. Plos One *7*, e26955.

Klanjscek, T., Nisbet, R.M., Priester, J.H., and Holden, P.A. (2013). Dynamic energy budget approach to modeling mechanisms of CdSe quantum dot toxicity. Ecotoxicology *22*, 319-330.

Koch, A.L. (1959). DEATH OF BACTERIA IN GROWING CULTURE. Journal of Bacteriology *77*, 623-629.

Koch, A.L. (1970). Turbidity measurements of bacterial cultures in some available commercial instruments. Analytical Biochemistry *38*, 252-259.

Krishnamurthi, V.R., Chen, J., and Wang, Y. (2019). Silver ions cause oscillation of bacterial length of Escherichia coli. Scientific Reports *9*, 11745.

Niyonshuti, II, Krishnamurthi, V.R., Okyere, D., Song, L., Benamara, M., Tong, X., Wang, Y., and Chen, J.Y. (2020). Polydopamine Surface Coating Synergizes the Antimicrobial Activity of Silver Nanoparticles. Acs Applied Materials & Interfaces *12*, 40067-40077.

Stevenson, K., McVey, A.F., Clark, I.B.N., Swain, P.S., and Pilizota, T. (2016). General calibration of microbial growth in microplate readers. Scientific Reports *6*, 38828.
